# Novel Trocars and Suspension System Application in Gasless Transoral Endoscopic Thyroidectomy Vestibular Approach Oral Endoscopic Surgery

**DOI:** 10.3389/fonc.2021.694133

**Published:** 2021-08-09

**Authors:** Jing Fang, Jianjun Liu, Xucai Zheng, Shengying Wang

**Affiliations:** ^1^Department of Head and Neck Surgery, West District of The First Affiliated Hospital of University of Science and Technology of China, Division of Life Sciences and Medicine, University of Science and Technology of China, Hefei, China; ^2^Department of Head and Neck Surgery, Anhui Provincial Cancer Hospital, Hefei, China

**Keywords:** trocar, TOETVA, NOTES, endoscopic surgery, thyroid cancer

## Abstract

In the current study, we reported our initial experience of gasless transoral endoscopic thyroidectomy vestibular approach (TOETVA) by novel trocars and a suspension system. Between February 2019 to September 2020, thyroid cancer patients with indicated central lymph node metastasis by imaging examination who had received gasless TOETVA by our designed trocars and suspension system in The First Affiliated Hospital of University of Science and Technology of China were reviewed. A total of 95 thyroid cancer patients that received gasless TOETVA were included in this analysis. Of note, 73 cases underwent one-sided lobectomy and the remaining 22 cases underwent total thyroidectomy. All thyroid cancer patients underwent central lymph node dissection (CND). The average total examined lymph nodes number was 8.55 ± 5.67 per individual. No serious complications occurred during or after the operation besides one patient who had a short-term recurrent laryngeal nerve (RLN) deficit and one patient who had delayed postoperative bleeding. In conclusion, the use of novel trocars and a suspension system can effectively improve the safety and efficacy of TOETVA.

## Background

As natural orifice transluminal endoscopic surgery (NOTES) technology has developed in recent years, NOTES has been the subject of increasing interest in thyroid surgery (1–4). The concept of the transoral endoscopic thyroidectomy vestibular approach (TOETVA) applied in thyroid surgery was first reported by Richmon et al. And nowadays, the TOETVA has been widely appreciated in thyroid surgery (5–10). Compared with other endoscopic surgical approaches, the oral hidden incision of TOETVA can be regarded as a real scar-free operation. Moreover, the shorter surgical path also makes the neck lymph nodes resection easier and safer than other approaches (10–12). Indeed, in the thyroid surgery field, TOETVA is the most cited technique in recent years.

Currently, carbon dioxide (CO_2_) insufflation is frequently used to maintain the working space in NOTES. However, the inherent limitation of CO_2_ insufflation should be recognition. It does increase the risk of CO_2_-related complications, such as CO_2_ embolism, pneumothorax, pneumomediastinum, and subcutaneous emphysema (13). In addition, it may cause tumor implantation and metastasis during the surgery (14). Therefore, a concept which uses the gasless method instead of CO_2_ insufflation in NOTES has been proposed. Since 2008, gasless trans-axillary endoscopic thyroid surgery has been reported, and there have been numerous studies regarding its safety and efficacy (15, 16). Researchers usually use robot-assisted technology in gasless NOTES, which requires higher clinical costs and a longer learning curve. However, due to the special characteristics of the oral vestibule, traditional laparoscopic surgical instruments are not suitable for gasless TOETVA, one of the difficult imports is instrument interference (17). In fact, only a few studies with small samples supported the safety and efficacy of gasless TOETVA (5, 18, 19).

In the past two years, we developed and performed a novel gasless TOETVA method that used novel trocars and a new suspension system designed by our team for thyroid cancer. A total of 95 thyroid cancers patients have successfully received gasless TOETVA with novel trocars and a new suspension system in our institution. In this prospective study, we report our initial experience and discuss the safety and efficacy of the novel redesigned trocars and the new suspension system.

## Subjects and Methods

### Patients

This study was performed in the Department of Head and Neck Surgery, The First Affiliated Hospital of University of Science and Technology of China. A total of 95 cases completely received gasless TOETVA between February 2019 to September 2020. All patients were diagnosed with thyroid cancer by fine needle aspiration cytology before surgery. Only the patients who were indicated to have central lymph node metastasis by imaging examination and received CND were selected. This study was approved by the hospital ethics committee.

In the current study, operative time was defined as the time from the initial skin incision to the point of final closure. Temporary hypoparathyroidism was defined by symptomatic hypocalcemia requiring calcitriol and calcium supplements in the immediate postoperative period. Permanent hypoparathyroidism was defined by low or undetectable PTH levels requiring calcitriol and calcium supplements ≥ 6 months from surgery. Temporary RLN deficit was identified by an independent laryngologist. Permanent RLN deficit was defined as a persistent deficit ≥ 6 months after surgery. The study was carried out with approval of the Institutional Review Board (IRB: NO.2019-21) of our hospital.

### Surgical Instruments

**Figure 1:**
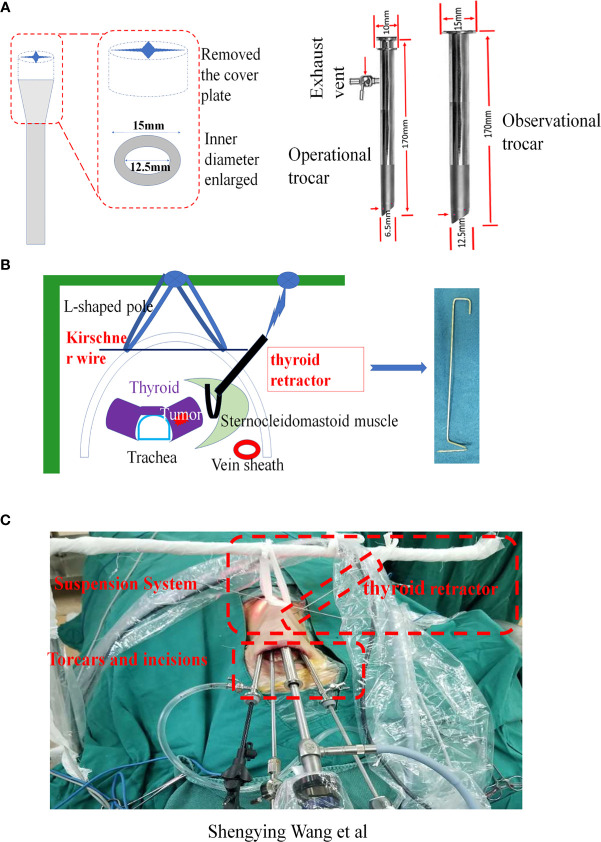
Application of the new trocars and suspension system. **(A)** The details of the new trocars. **(B)** The details of suspension system. **(C)** Gasless TOETVA using the new trocars and suspension system (view from outside).

The special instruments for gasless TOETVA included three self-designed trocars (two operational trocars, one observational trocar) and two 1.5mm Kirschner wires. These uniquely designed trocars had successfully obtained China’s medical device patent approval. The novel trocars and suspension system were designed as shown in **Figures 1A, B**. Briefly, we removed the antileak valve of the traditional trocar and reduced the outer diameter of the trocars. In addition, the inner diameters of the trocars were also increased. Undoubtedly, the self-redesigned trocars can significantly reduce the interference between the instruments. The larger entrance size and inner diameter also can speed up the air circulation, which can facilitate the eliminate of smoke during the operation. The suspension system included two Kirschner wires. One of the Kirschner wires was bent into a hook shape and named thyroid retractor.

### Surgical Methodology

The patient was placed in the supine position with the neck extended. The trachea was intubated through the mouth for anesthesia. The tracheal tube was fixed on one side of the mouth. After routine painting and draping, the oral cavity was disinfected again by iodophor before surgery. A disinfected L-shaped pole was wrapped by a sterile protective cover and then fixed on the side of the head as a suspension frame (**Figure 1C**). The surgeon stood on the side of the patient’s head, and the assistant was located on the right side of the surgeon.

**Figure 2:**
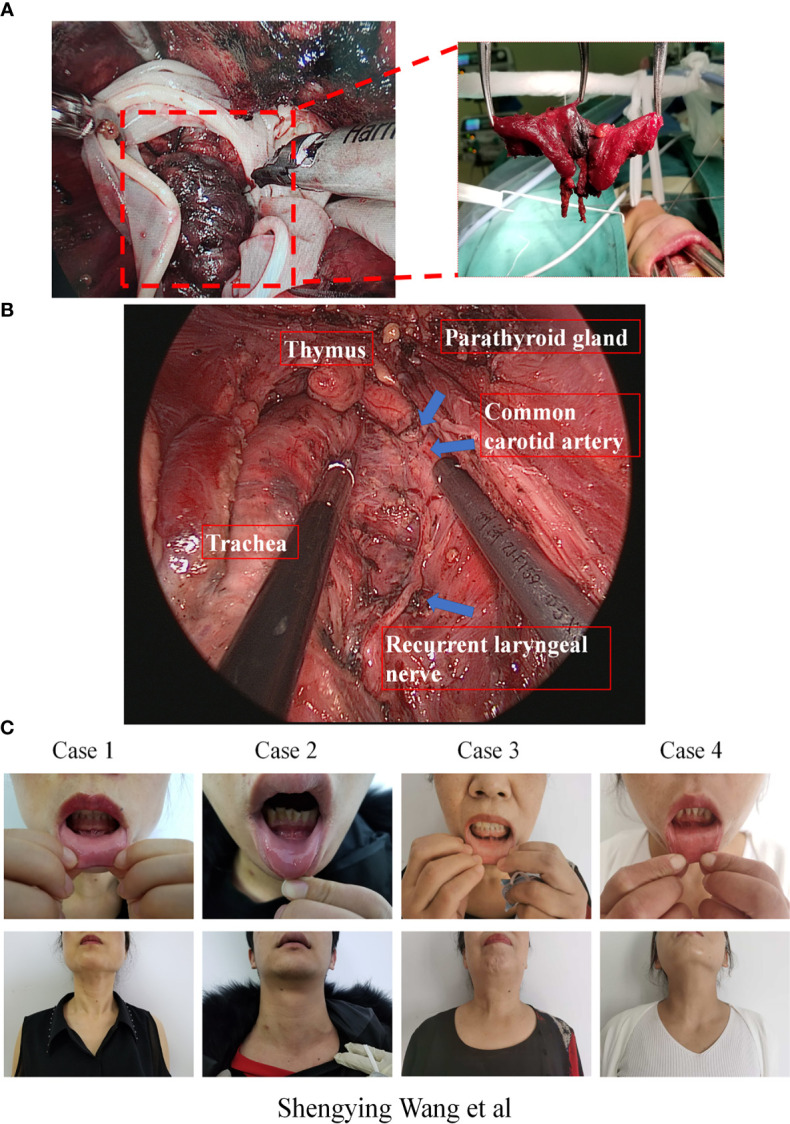
**(A)** The en bloc resected thyroid tissue and central lymph nodes are put into a special specimen retrieval bag and then moved outside the body through the observation incision. **(B)** Important organs view after the specimen resection during gasless TOETVA. **(C)** Patients’ front view of oral vestibule incisions and neck after surgery.

The surgery was performed in two steps. Step one: Establish the operation space. Firstly, before surgery, we injected an epinephrine-containing swelling with normal saline fluid (1:500,000 epinephrine) around the oral vestibule to reduce bleeding from the incision. Then, three incisions were separately cut through the oral vestibule. One 2 cm sized middle midline curvilinear incision in the vestibule was made for the observation port, and two 0.6 cm sized curvilinear incisions on each side of the midline curvilinear incision were made for the operation ports. The observational port was located in front of the lower lip frenulum and 5 mm from the gingival root, and the two operational ports on both sides were located at the buccal mucosa of the first premolars on both sides. Then, the subcutaneous space of the neck was separated through the observation port with a hypodermic peeling bar to the prebuild operating space. To ensure the surgical space was clear, one of the operating port trocars (usually the operating port where the ultrasonic knife was placed) was connected to a vacuum to maintain the operation space with negative pressure, and the gap between the observational port trocar and the endoscope lens and another trocar was used to inhale air to facilitate air circulation (**Figure 1C**). The endoscopic operative space was expanded step by step. The separation range of the neck flaps was down to the suprasternal fossae, and both sides were up to the front edge of the sternocleidomastoid muscle. As the space expanded enough for surgery, a Kirshner wire could penetrate the skin on the surface of the thyroid and the two ends of the Kirshner wire were fixed on the L-shaped pole above the patient’s neck with a bandage suspension. Step two, after the operation space was established, the surgery was performed as follows. Firstly, to quickly eliminate surgical smoke, an additionally vacuum tube was placed next to the observation port to keep the operation space under negative pressure. Secondly, the strap muscle of the neck was dissociated to expose the thyroid gland. A self-designed thyroid retractor was inserted into the medial edge of the sternocleidomastoid muscle and fixed in the head frame. The operation space was maintained by the Kirshner wire and thyroid retractor (**Figure 1C**). Then, the upper pole of the thyroid was slightly separated from the cricothyroid space. The anterior Delphian lymph nodes were swept and the vertebral thyroid lobe was removed. Lastly, before removal of the specimen, the surgeon distinguished the superior laryngeal nerve, recurrent laryngeal nerve, and parathyroid gland. The thyroid and central lymph nodes were completely removed, and the organs were kept at the original position at the same time (**Figure 2**). After the endoscopic procedures were completed, a negative pressure drainage tube remained in the surgical space. Postoperative management followed according to the routine TOETVA.

## Results

### Patients’ Clinical Characteristics

As shown in **Table 1**, a total of 95 thyroid cancer patients received gasless TOETVA. There were 7 male patients and 88 female patients in this study. The average age was 34.32 ± 8.83 years old, and the average tumor diameter was 0.87 ± 0.66 cm. All patients successfully completed the operation without transfer to traditional surgery. Only one patient had delayed bleeding on the 7th day after surgery, and one patient had a hoarse voice after surgery and recovered after one month.

**Table 1:** Patients characteristics (n = 95).

	Median	Range/Percent
Age (year)	34.32 ± 8.83	22-64
Sex		
Male	7	7.40%
Female	88	92.60%
Location		
Right	45	47.40%
Left	35	36.80%
Isthmus	5	5.30%
Bilateral	10	10.50%
Tumor size (cm)	0.87 ± 0.66	0.10-4.20
PLN	1.37 ± 2.29	0-14
TLN	8.55 ± 5.67	1-30
Operation time (min)	194.14 ± 43.13	113-305
Extent of surgery		
Total	22	23.20%
Lobectomy	73	76.80%
Drain removal (days)	6.50	3-11

PLN, positive lymph node; TLN, total examined lymph node.

## Discussion

Since the first prospective proof-of-concept TOEVTA successfully performed by Wilhelm et al. in 2009, it has gradually become popular in thyroid cancer patient treatment (4, 7). However, after lots of operations performed in clinical settings, increasing problems have also been found and reported. Firstly, compared with other NOTES approaches, maintaining the operating space in TOETVA is harder because of the continuous tension of platysma. Secondly, given the abundance of blood vessels in the oral mucosa, CO_2_ is easily absorbed through the oral mucosa into blood and leads to related complications (13). In addition, endoscopic surgery supported by CO2 is considered to cause tumor implantation and metastasis during the surgery. Indeed, the CO_2_ insufflation method in thyroid endoscopic surgery hides a higher risk. Therefore, surgeons are beginning to explore an alternative method. Disappointingly, the progress of the alternative method in TOETVA is relatively slow. At present, only a few reports have focused on the gasless TOETVA. In 2013, Akihiro Nakajo and colleagues first reported eight thyroid disease patients who received gasless TOETVA in their institution (8). In this report, they created a new suspension method instead of gasless TOETVA, in which Kirschner wires with a diameter of 1.2 mm were inserted horizontally into the subplatysmal layer of the anterior cervical area to maintain a working space during the operation. In fact, this method is inconvenient for widespread application. The operation was performed through a single port in the oral vestibule. Given the intrinsic limitation of single-port surgery, surgical instruments easily interfere with each other during operation. Moreover, a small incision with small inner trocars also limited smoke elimination, which decreased the clearance of the operation area. In 2019, Yoon Woo Koh et al. reported 15 thyroid disease patients who received a modified gasless TOETVA in Korea (18). They used a sling retractable blade which was inserted into the neck subcutaneously to maintain the operating space through the oral vestibular. Although this method reduces the instrumental interference of single-port surgery, it also has several drawbacks. For example, the retractable blade is placed through the observation port which restricts the use of the wider trocar during operation, it is therefore difficult to use the 10 mm lens which can ensure a wider surgical field during surgery. Moreover, due to the small size of the oral vestibule, the larger bases of traditional trocars are likely to interfere with each other during operation. In addition, to ensure the surgical space is clear, the smoke generated during the operation should be removed as soon as possible. However, due to low pressure insufflation and slow gas circulation, it is hard to effectively and timely remove the smoke during the operation. It reduces the clarity of the surgical space. Therefore, it is confirmed that inconvenient traditional laparoscopic surgical instruments are not suitable for gasless TOETVA. Novel convenient instruments for gasless TOETVA are urgently needed.

To ensure the gasless TOETVA is performed successfully, two problems should be resolved during the surgery. Firstly, it is necessary to maintain operating space stability. Secondly, it is necessary to eliminate the smoke which is generated from the surgery quickly. We have found that it is not necessary to keep the operation space sealed. Thus, we first attempted to develop an improved gasless TOETVA method with newly designed trocars and suspension system. We removed the antileak valve of the traditional trocar base, and the reduced outer diameter of the trocars can not only reduce the interference of the instruments during surgery, but can also accelerate air circulation and improve the clarity of the operation space. In addition, we established a new method in which the surgical space was maintained by a new suspension system. Indeed, the novel trocars can speed up air circulation, which can facilitate the removal of smoke during the operation. The suspension system can provide a more stable and wider operation area. As a result, we found the above problems were effectively and safely solved.

Besides of the trocars, our suspension system was also superior to that of previous reports. Firstly, our suspension system does not need to occupy the observational incision. Therefore, a larger inner trocar and endoscope can be used in the operation. Secondly, our suspension has less damage, especially in the submental area, which can significantly improve the sensation of the submental area and lip after surgery. Thirdly, we first added a thyroid retractor into the gasless TOETVA, which can pull the sternocleidomastoid muscle away from thyroid and expose the side of the thyroid gland more effectively. It can facilitate the exposure and protect of the parathyroid and recurrent laryngeal nerve during surgery. Thus, the suspension unit is usefully used for en bloc CND. The redesigned trocars and suspension system can maintain clearly and stably the operation area. Indeed, the average operation time in our study is shorter than that of previous studies. Although all cases in this study received CND, the average surgery time in our study was 194.14 ± 43.13 min, which was still significantly shorter than that of the previously study (361 min in Nakajo et al. study). In addition, the number of totally resected lymph nodes was 8.55, which was significantly higher than that of the previously study (3 lymph nodes in Yoon Woo Koh et al. study). The increased number of lymph nodes removed can not only mitigate the risk of recurrence, but also help to effectively assess tumor burden (20, 21). The adequacy lymph node stage could provide an personalized recommendation for adjuvant radioactive iodine and surveillance intensity after surgery (22). We hypothesis that gasless TOETVA by our instruments, not only can significantly reduce the operation time and postoperative complications, but also can reduce the risk of local recurrence. In addition, we found this method can be applied to more types of thyroid disease surgery. Indeed, we confirmed that the novel trocars and suspension system can be used in gasless endoscopic assisted lateral neck lymph node dissection (**Supplementary Figure 1**).

In conclusion, the use of the improved trocars and suspension system for gasless TOETVA can not only eradicate the related complications caused by CO_2_ insufflation, but can also reduce the interference of instruments and improve the clarity of the operation space. The redesigned trocars and suspension system seem to be safe and feasible, which can replace the traditional trocar and suspension system in future.

## Data Availability Statement

The original contributions presented in the study are included in the article/**Supplementary Material**. Further inquiries can be directed to the corresponding author.

## Ethics Statement

The studies involving human participants were reviewed and approved by Anhui tumor Hospital ethics committee. The patients/participants provided their written informed consent to participate in this study.

## Author Contributions

Conceptualization: JF and SW. Collection and assembly of data: JF and JL. Data analysis and interpretation: JF and JL. Writing: JF and JL. Visualization: SW. Supervision: SW. Project administration: SW. All authors contributed to the article and approved the submitted version.

## Funding

National Natural Science Foundation of China, Grant/Award Number: 81802641.

## Conflict of Interest

The authors declare that the research was conducted in the absence of any commercial or financial relationships that could be construed as a potential conflict of interest.

## Publisher’s Note

All claims expressed in this article are solely those of the authors and do not necessarily represent those of their affiliated organizations, or those of the publisher, the editors and the reviewers. Any product that may be evaluated in this article, or claim that may be made by its manufacturer, is not guaranteed or endorsed by the publisher.

## Supplementary Material

Supplementary Figure 1 Important organs view after gasless endoscopic assisted lateral neck lymph node resection.
