# Corrigendum: Novel Trocars and Suspension System Applicated in Gasless Transoral Endoscopic Thyroidectomy Vestibular Approach Oral Endoscopic Surgery

**DOI:** 10.3389/fonc.2021.760942

**Published:** 2021-09-20

**Authors:** Jing Fang, Jianjun Liu, Xucai Zheng, Shengying Wang

**Affiliations:** ^1^Department of Head and Neck Surgery, West District of The First Affiliated Hospital of University of Science and Technology of China, Division of Life Sciences and Medicine, University of Science and Technology of China, Hefei, China; ^2^Department of Head and Neck Surgery, Anhui Provincial Cancer Hospital, Hefei, China

**Keywords:** trocar, TOETVA, NOTES, endoscopic surgery, thyroid cancer

In the original article, there was a mistake in ***Table 1*** as published. The range of PLN number was wrong. The corrected ***Table 1*** appears below.

**Table 1:** Patients characteristics (n = 95).

	Median	Range/Percent
Age (year)	34.32 ± 8.83	22-64
Sex		
Male	7	7.40%
Female	88	92.60%
Location		
Right	45	47.40%
Left	35	36.80%
Isthmus	5	5.30%
Bilateral	10	10.50%
Tumor size (cm)	0.87 ± 0.66	0.10-4.20
PLN	1.37 ± 2.29	0-14
TLN	8.55 ± 5.67	1-30
Operation time (min)	194.14 ± 43.13	113-305
Extent of surgery		
Total	22	23.20%
Lobectomy	73	76.80%
Drain removal (days)	6.50	3-11

PLN, positive lymph node; TLN, total examined lymph node.

In the original article, there was a misspelled word, the number of observational trocar should be one. A correction has been made to ***Subjects and Methods***, ***Surgical Instruments***, ***paragraph 1***:

“The special instruments for gasless TOETVA included three self-designed trocars (two operational trocars, one observational trocar) and two 1.5mm Kirschner wires. These uniquely designed trocars had successfully obtained China’s medical device patent approval. The novel trocars and suspension system were designed as shown in **Figures 1A, B**. Briefly, we removed the antileak valve of the traditional trocar and reduced the outer diameter of the trocars. In addition, the inner diameters of the trocars were also increased. Undoubtedly, the self-redesigned trocars can significantly reduce the interference between the instruments. The larger entrance size and inner diameter also can speed up the air circulation, which can facilitate the eliminate of smoke during the operation. The suspension system included two Kirschner wires. One of the Kirschner wires was bent into a hook shape and named thyroid retractor.”

Additionally, we used the result from earlier data in the discussion, and the percentage of cases given as having received CND was wrong, it should have been all. A correction has been made to ***Discussion***, ***paragraph 3***:

“Besides of the trocars, our suspension system was also superior to that of previous reports. Firstly, our suspension system does not need to occupy the observational incision. Therefore, a larger inner trocar and endoscope can be used in the operation. Secondly, our suspension has less damage, especially in the submental area, which can significantly improve the sensation of the submental area and lip after surgery. Thirdly, we first added a thyroid retractor into the gasless TOETVA, which can pull the sternocleidomastoid muscle away from thyroid and expose the side of the thyroid gland more effectively. It can facilitate the exposure and protect of the parathyroid and recurrent laryngeal nerve during surgery. Thus, the suspension unit is usefully used for en bloc CND. The redesigned trocars and suspension system can maintain clearly and stably the operation area. Indeed, the average operation time in our study is shorter than that of previous studies. Although all cases in this study received CND, the average surgery time in our study was 194.14 ± 43.13 min, which was still significantly shorter than that of the previously study (361min in Nakajo et al. study). In addition, the number of totally resected lymph nodes was 8.55, which was significantly higher than that of the previously study (3 lymph nodes in Yoon Woo Koh et al. study). The increased number of lymph nodes removed can not only mitigate the risk of recurrence, but also help to effectively assess tumor burden (20, 21). The adequacy lymph node stage could provide an personalized recommendation for adjuvant radioactive iodine and surveillance intensity after surgery (22). We hypothesis that gasless TOETVA by our instruments, not only can significantly reduce the operation time and postoperative complications, but also can reduce the risk of local recurrence. In addition, we found this method can be applied to more types of thyroid disease surgery. Indeed, we confirmed that the novel trocars and suspension system can be used in gasless endoscopic assisted lateral neck lymph node dissection (**Supplementary Figure 1**).”

The authors apologize for these errors and state that this does not change the scientific conclusions of the article in any way. The original article has been updated.

## Publisher’s Note

All claims expressed in this article are solely those of the authors and do not necessarily represent those of their affiliated organizations, or those of the publisher, the editors and the reviewers. Any product that may be evaluated in this article, or claim that may be made by its manufacturer, is not guaranteed or endorsed by the publisher.

