# Supplementary figures and images for: Novel Trocars and Suspension System Application in Gasless Transoral Endoscopic Thyroidectomy Vestibular Approach Oral Endoscopic Surgery
Source: Front Oncol. 2021 Aug 9;11:694133. doi: 10.3389/fonc.2021.694133 (PMC8416175; doi:10.3389/fonc.2021.694133)

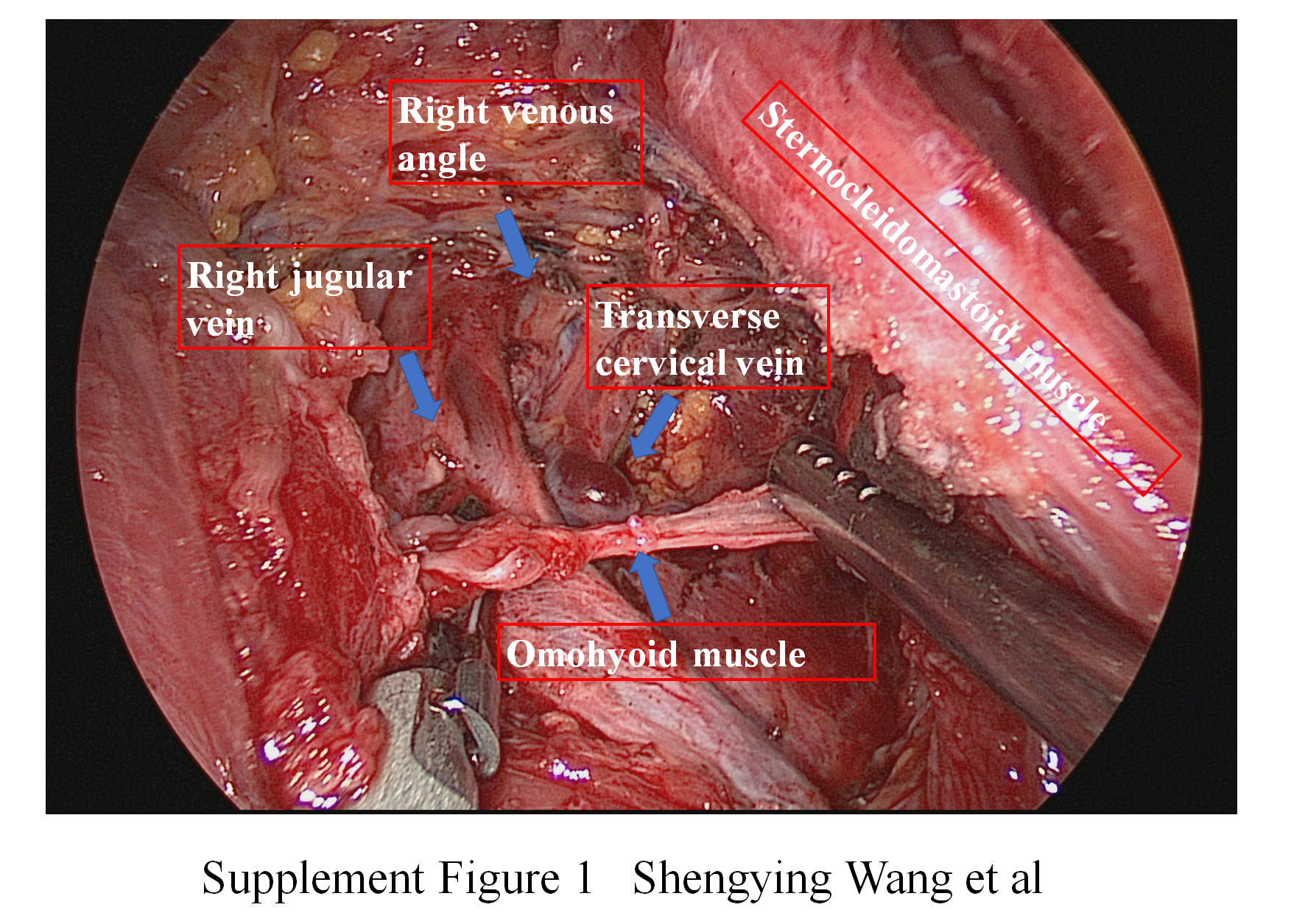

Supplement: Supplementary Figure 1 — Important organs view after gasless endoscopic assisted lateral neck lymph node resection. [file Image_1.tif]
